# Finding Lung-Cancer-Related lncRNAs Based on Laplacian Regularized Least Squares With Unbalanced Bi-Random Walk

**DOI:** 10.3389/fgene.2022.933009

**Published:** 2022-07-22

**Authors:** Zhifeng Guo, Yan Hui, Fanlong Kong, Xiaoxi Lin

**Affiliations:** Department of Oncology, Chifeng Municipal Hospital, Chifeng, China

**Keywords:** lung cancer, lncRNA, biomarker, lncRNA-disease association, laplacian regularized least squares, unbalanced bi-random walk

## Abstract

Lung cancer is one of the leading causes of cancer-related deaths. Thus, it is important to find its biomarkers. Furthermore, there is an increasing number of studies reporting that long noncoding RNAs (lncRNAs) demonstrate dense linkages with multiple human complex diseases. Inferring new lncRNA-disease associations help to identify potential biomarkers for lung cancer and further understand its pathogenesis, design new drugs, and formulate individualized therapeutic options for lung cancer patients. This study developed a computational method (LDA-RLSURW) by integrating Laplacian regularized least squares and unbalanced bi-random walk to discover possible lncRNA biomarkers for lung cancer. First, the lncRNA and disease similarities were computed. Second, unbalanced bi-random walk was, respectively, applied to the lncRNA and disease networks to score associations between diseases and lncRNAs. Third, Laplacian regularized least squares were further used to compute the association probability between each lncRNA-disease pair based on the computed random walk scores. LDA-RLSURW was compared using 10 classical LDA prediction methods, and the best AUC value of 0.9027 on the lncRNADisease database was obtained. We found the top 30 lncRNAs associated with lung cancers and inferred that lncRNAs TUG1, PTENP1, and UCA1 may be biomarkers of lung neoplasms, non-small–cell lung cancer, and LUAD, respectively.

## 1 Introduction

Cancers are posing threat for the health of humans (Yang et al., 2013; Liu et al., 2021). Lung cancer is the most common cancer worldwide and one of the leading causes of cancer-relevant deaths, and it has been so for many years. Thus, in 2008, the global statistical analysis demonstrated that approximately 1.6 million new lung cancer cases were diagnosed, and 1.4 million deaths were confirmed globally. In 2012, there were 1.8 million of new lung cancer diagnoses and 1.6 million deaths (de Groot et al., 2018; Howlader et al., 2020). In 2018, the number of new lung cancer cases exceeded 2 million and the number of deaths exceeded 1.7 million (Yuan et al., 2019). In the United States, approximately 234,000 cases of lung cancer were diagnosed the same year. This year, lung cancer diagnosis account for 14 and 13% of new cases in men and women, respectively. Estimation of mortality is 83,550 and 70,500 deaths in men and women, respectively. Lung carcinoma is one of cancers with the lowest survival rate. It is usually not diagnosed until an advanced stage (de Groot et al., 2018; Howlader et al., 2020).

Despite the fast development of lung cancer therapy, high morbidity and mortality rates still pose a severe challenge for cancer researchers. The majority of patients with advanced-stage lung cancer have been ultimately poorly diagnosed. Thus, designing efficient therapy strategies is extremely important for lung cancer patients. However, existing techniques applied to diagnosis and therapies of lung cancer remain suboptimal. Thus, better strategies supplementing or replacing the existing techniques are urgent. Genome-wide association studies have found numerous genetic variants relevant to various cancers, one-third of which are densely linked to noncoding regions. The noncoding RNAs can be used as biomarkers of lung cancers. Therefore, accurate biomarker identification is urgently required to effectively diagnose lung cancer and boost the survival rate while decreasing its mortality and morbidity (Huang et al., 2017; Roointan et al., 2019; Yang et al., 2020).

Long noncoding RNAs (lncRNAs) are a type of noncoding RNAs that has over 200 nucleotides and post-transcriptional modifications including splicing, capping, and polyadenylation. lncRNAs can be used as a guide for protein-DNA interactions, protein-RNA interactions, and protein–protein interactions (Peng et al., 2020a). With the fast advancement of cancer genomics, many lncRNAs have been demonstrated to be aberrantly expressed in diverse cancers and play key action in the development of tumors through modulation of cancer-related signaling pathways. lncRNAs can regulate survival, metastasis, angiogenesis, and proliferation of tumor cells. Therefore, lncRNAs can be used as potential biomarkers and therapeutic targets in cancers by interacting with proteins (Chandra Gupta and Nandan Tripathi, 2017). For example, Peng et al. and her groups (Peng et al., 2021a; Zhou L. Q. et al., 2021; Peng et al., 2021b; Zhou L. et al., 2021; Tian et al., 2021; Peng et al., 2022) designed a series of state-of-the-art lncRNA-protein interaction prediction methods and significantly improved biomarker identification for various diseases. In addition, lncRNA SNHG14, BCRT1, DSCAM-AS1, MaTAR24, and HOTAIR have been validated to densely link to breast cancer (Niknafs et al., 2016; Dong et al., 2018; Chang et al., 2020; Liang et al., 2020; Yang et al., 2022; Xue et al., 2016). HOTAIR has been reported to be highly expressed in non-small–cell lung cancer (NSCLC) and affect NSCLC tumorigenesis and metastasis. In addition, many biomarkers (for example, CA125, NSE, CEA, VEGF, and EGFR (Khanmohammadi et al., 2020) have been validated to associate with lung cancer.

More importantly, many machine learning methods, especially deep-learning methods, have been applied to identify lncRNA biomarkers of various diseases through lncRNA-disease association prediction. Thus, Fan et al. (2022) designed an LDA prediction method (GCRFLDA) using the graph convolutional matrix completion. Ma Y (Ma, 2022) exploited a deep multi-network embedding-based LDA inference framework. Wu et al. (2021) integrated graph auto-encoder and random forest for LDA prediction. Sheng et al. (2021) developed an attentional multi-level representation encoding method to find new LDAs combining convolutional and variance autoencoders. Zhao et al. (2022) proposed a heterogeneous graph attention network-based LDA identification model. These methods significantly improved the LDA prediction.

With the development of single cell RNA sequencing technologies (Peng et al., 2020b), we can obtain numerous RNA data. These data can improve the analyses of RNA data, for example, SARS-CoV-2 (Xu et al., 2020; Li et al., 2021). By finding new lncRNA biomarkers, we can design corresponding therapeutic strategies for lung cancer based on drug repositioning (Peng et al., 2015; Liu et al., 2020; Meng et al., 2022; Shen et al., 2022).

Although experimental methods found a few biomarkers for lung cancer, they are time-consuming and waste of resources. Therefore, computational techniques have been exploited to infer potential biomarkers for lung cancer. However, the majority of computational approaches need to improve the inference performance. In this study, to analyze the diagnostic, prognostic, and therapeutical potential of lncRNAs in lung cancer patients, we exploit a computational model combining Laplacian regularized least square and unbalanced bi-random walk, LDA-RLSURW, to predict possible lncRNA biomarkers for lung cancer.

## 2 Datasets

First, the lncRNA-disease association dataset was collected. The dataset can be obtained from the lncRNADisease database at http://www.cuilab.cn/lncrnadisease (Chen et al., 2012). We obtained 82 lncRNAs, 157 diseases, and 701 associations after excluding lncRNAs without record in the lncRNADisease database and diseases with inappropriate names or without MeSH tree numbers.

## 3 Methods

**FIGURE 1:**
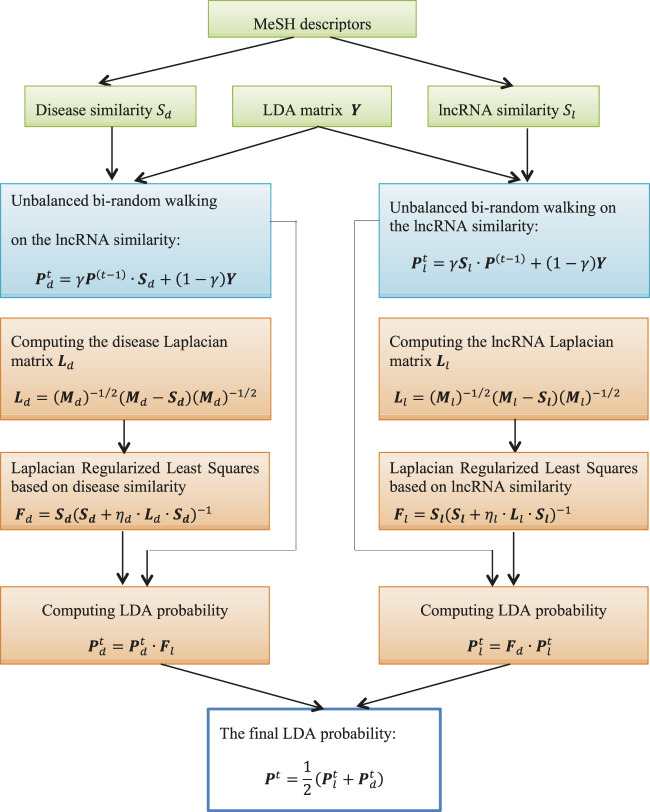
Flowchart of LDA-RLSURW.

This study developed an lncRNA-disease association prediction method LDA-RLSURW. First, LDA-RLSURW computed disease semantic similarity and lncRNA functional similarity. Second, LDA-RLSURW calculated the initial association probability of each lncRNA-disease pair using unbalanced bi-random walk based on disease similarity matrix and lncRNA similarity, respectively. In conclusion, the computed initial lncRNA-disease association probabilities were further updated Laplacian regularized least squares. The flowchart of LDA-RLSURW is presented in Figure 1.

### 3.1 Disease Semantic Similarity

Semantic similarity between diseases can be computed using the directed acyclic graph (DAGs) based on their MeSH descriptors (Fan et al., 2020). Given a disease A , let its DAG be represented as $DAG_{A}=\left\{T_{A},E_{A}\right\}$ , where $T_{A}$ denotes the ancestor node set of A including A , and $E_{A}$ denotes all edge set. For a disease term $t\in T_{A}$ in $DAG_{A}$ , its semantic contribution to A can be computed by Eq. 1 provided by LNCSIM1 (Chen et al., 2015):

$$SV_{A}^{1}\left(t\right)=\begin{array}{cc} 1 & t=A\\ \text{max}(\alpha \times SV_{A}^{1}\left(t^{\prime }\right){\mathrm{|t}}^{\prime }\in C\left(t\right) & t\neq A \end{array}\;,$$ (1)

where C(t) denotes the children of t and α denotes a sematic contribution value of an edge linking $t^{\prime }$ to t in $E_{A}$ .

In Eq. 1, we assume that terms at one identical layer from $DAG_{A}$ have identical semantic contribution to A . However, when terms $t_{1}$ and $t_{2}$ are in the identical layer of $DAG_{A}$ , and $t_{1}$ appears less than $t_{2}$ in $DAG_{A}$ , the results from $t_{1}$ may be more specific than $t_{2}$ . Thus, it could be more reasonable that $SV_{A}^{1}\left(t_{1}\right)$ is larger than $SV_{A}^{1}\left(t_{2}\right).$

Considering this situation, we compute another semantic contribution value for disease A by Eq. 2 provided by LNCSIM1 (Chen et al., 2015):

$$SV_{A}^{2}\left(t\right)=-log\frac{Dags\left(t\right)}{D},$$ (2)

where D denotes the number of all diseases in the MeSH database and Dags(t) denotes the number of DAG s, including the disease term t . In conclusion, the semantic contribution value of disease A in $DAG_{A}$ can be computed by

$$SV_{A}^{3}\left(t\right)=\begin{array}{cc} 1 & t=A\\ \text{max}(\left(\alpha +\beta \right)SV_{A}^{3}\left(t^{\prime }\right){\mathrm{|t}}^{\prime }\in C\left(t\right) & t\neq A \end{array},$$ (3)

where β denotes the information content contribution factor, and

$$\beta =\frac{\underset{k\in K}{\max}\left(Dags\left(k\right)\right)-dags\left(t\right)}{D},$$ (4)

where K denotes the disease set from the MeSH database.

Thus, the contribution of all diseases in $DAG_{A}$ to A can be represented as

$$SV\left(A\right)=\underset{t\in T_{A}}{\sum }SV_{A}^{3}\left(t\right).$$ (5)

In summary, the semantic similarity between diseases *A* and *B* can be computed by Eq. 6:

$$S_{d}\left(A,B\right)=\frac{\sum_{t\in T_{A}\cap T_{B}}\left(SV_{A}^{3}\left(t\right)+SV_{B}^{3}\left(t\right)\right)}{SV\left(A\right)+SV\left(B\right)}.$$ (6)

### 3.2 lncRNA Functional Similarity

We calculate the lncRNA similarity using the approach provided by Fan et al. (2020). Assuming that *DG(u)*/*DG(v)* denotes diseases associated with lncRNA u / v  based on the LDA matrix, the lncRNA similarity between u and v was computed through semantic similarity between diseases involved in *DG(u)* and *DG(v)*. First, we construct a disease semantic similarity sub-matrix, where both rows and columns denote all diseases involved in *DG(u)*∪*DG(v)*, and the value of each element can be measured using the semantic similarity between corresponding diseases. Second, let $d_{u}$ / $d_{v}$ denote one disease in *DG(u)*/*DG(v)*; the similarity between $d_{u}$ / $d_{v}$ and *DG(v)*/*DG(u)* can be computed by Eqs. 7 and 8:

$$S\left(d_{u},DG\left(v\right)\right)=\underset{d\in DG\left(v\right)}{\max}\left(S_{d}\left(d_{u},d\right)\right),$$ (7)

$$S\left(d_{v},DG\left(u\right)\right)=\underset{d\in DG\left(u\right)}{\max}\left(S_{d}\left(d_{v},d\right)\right).$$ (8)

Third, the similarity between *DG(u)* to *DG(v)* and one between *DG(v)* to *DG(u)* can be calculated by Eqs. 9 and 10:

$$S_{u\to v}=\underset{d\in DG\left(u\right)}{\sum }S\left(d,DG\left(v\right)\right)\;,$$ (9)

$$S_{v\to u}=\underset{d\in DG\left(v\right)}{\sum }S\left(d,DG\left(u\right)\right).$$ (10)

In conclusion, the similarity between two lncRNAs u and v can be computed by Eq. 11:

$$S_{l}\left(u,v\right)=\frac{S_{u\to v}+S_{v\to u}}{\left|DG\left(u\right)\right|+\left|DG\left(v\right)\right|},$$ (11)

where |DG(u)|/|DG(v)| indicates the number of diseases in DG(u)/DG(v) .

### 3.3 Unbalanced Bi-Random Walk

In this section, inspired by Shen et al. (2022), we consider that the lncRNA similarity network and the disease network and design an unbalance bi-random walk model to score lncRNA-disease pairs. The two networks exhibit different topological structures. Therefore, we use different optimal walking step sizes when randomly walking on these two networks. That is, we propose an unbalanced bi-random walk algorithm. First, we compute lncRNA-disease association scores by randomly walking with the maximal iteration number of $n_{l}$ on the lncRNA network based on the lncRNA similarity by Eq. 12:

$$P_{l}^{t}=\gamma S_{l}\cdot P^{\left(t-1\right)}+\left(1-\gamma \right)Y\;\;\;for\;\;\;\;t=n_{l}.$$ (12)

In Eq. 12, at each step, the lncRNA similarity is fused with the random walk step by multiplying $S_{l}$ on the left of the lncRNA-disease association probability matrix. γ∈(0,1) is used to decrease the importance of circular bigraphs where the paths are longer during random walk and balance possible and known LDAs.

Second, we compute lncRNA-disease association scores by randomly walking with the maximal iteration number of $n_{d}$ on the disease network based on the disease similarity by Eq. 13:

$$P_{d}^{t}=\gamma P^{\left(t-1\right)}\cdot S_{d}+\left(1-\gamma \right)Y\;for\;t=n_{r}.$$ (13)

In Eq. 13, at each step, disease similarity is fused with the random walk step by multiplying $S_{d}$ on the right of the lncRNA-disease association probability matrix.

### 3.4 Laplacian Regularized Least Squares

In the last section, we compute the association probability for each lncRNA and disease using unbalanced bi-random walk method. However, for the algorithm, the jump condition is determined by known LDA data and the two similarity matrices. For a node $n_{i}$ in an LDA network, if two other nodes $n_{j}$ and $n_{k}$ exhibit the same similarity with $n_{i}$ , $n_{j}$ and $n_{k}$ may equally contribute to the jump. However, the node that has lower similarities with other nodes should have more contribution. Thus, we introduce Laplacian regularized least squares to solve the problem. First, the lncRNA Laplacian matrix $L_{l}$ and the disease Laplacian matrix $L_{d}$ are normalized to assess the jump probability for each node *via* Eqs 14, 15.

$$L_{l}={\left(M_{l}\right)}^{-1/2}\left(M_{l}-S_{l}\right){\left(M_{l}\right)}^{-1/2},$$ (14)

$$L_{d}={\left(M_{d}\right)}^{-1/2}\left(M_{d}-S_{d}\right){\left(M_{d}\right)}^{-1/2},$$ (15)

where $M_{l}/M_{d}$ represent the diagonal matrices of lncRNAs/diseases whose element $M_{l}\left(i,i\right)/M_{d}\left(j,j\right)$ denotes the summation of the i -th/ j -th row of $S_{l}/S_{d}$ .

Second, to optimize the above minimum problems, the loss functions in the lncRNA and disease spaces are defined based on Laplacian matrices $L_{l}$ and $L_{d}$ *via* Eqs. 11 and 12, respectively:

$$\underset{F_{l}}{\min}\left[{\|Y^{T}-F_{l}\|}_{F}^{2}+\eta_{l}{\|F_{l}\cdot L_{l}\cdot {\left(F_{l}\right)}^{T}\|}_{F}^{2}\right],$$ (16)

$$\underset{F_{d}}{\min}\left[{\left\|Y-F_{d}\right\|}_{F}^{2}+\eta_{d}{\|F_{d}\cdot L_{d}\cdot {\left(F_{d}\right)}^{T}\|}_{F}^{2}\right]\;,$$ (17)

where $\;{\|\;\cdot \|}_{F}$ denotes the Frobenius norm, ${\left(\cdot \right)}^{T}$ indicates the transpose, and $\eta_{v}$ and $\eta_{d}$ represent trade-off parameters. Models (11) and (12) can be solved *via* Eqs. 13 and 14, respectively:

$$F_{l}^{*}=S_{l}{\left(S_{l}+\eta_{l}\cdot L_{l}\cdot S_{l}\right)}^{-1}Y^{T},$$ (18)

$$F_{d}^{*}=S_{d}{\left(S_{d}+\eta_{d}\cdot L_{d}\cdot S_{d}\right)}^{-1}Y.$$ (19)

To comprehensively detect the effect of unbalanced bi-random walk on the inference performance, we replace Y using LDA association probabilities computed by random walks. Assume that Eqs. 20 and 21 can be defined as follows:

$$F_{l}=S_{l}{\left(S_{l}+\eta_{l}\cdot L_{l}\cdot S_{l}\right)}^{-1},$$ (20)

$$F_{d}=S_{d}{\left(S_{d}+\eta_{d}\cdot L_{d}\cdot S_{d}\right)}^{-1}.$$ (21)

At the t -th walking, Eqs. 22 and 23 can be defined as

$$P_{l}^{t}=F_{d}\cdot P_{l}^{t},$$ (22)

$$P_{d}^{t}=P_{d}^{t}\cdot F_{l}.$$ (23)

In conclusion, the LDA-RLSURW calculates the association score for each lncRNA-disease pair by combining association scores from the lncRNA and disease networks using Eq. 24:

$$P^{t}=\frac{1}{2}\left(P_{l}^{t}+P_{d}^{t}\right).$$ (24)

## 4 Experiments

### 4.1 Experimental Settings and Evaluation

The semantic contribution weight α is set as 0.5, the jump probability γ is set as 0.001, the maximal iteration number on the lncRNA network $n_{l}$ is set as 31, the maximal iteration number on the disease network $n_{r}$ is set as 1, and Laplacian regularized least square parameters $\eta_{l}$ and $\eta_{d}$ are set as 0.01. When the parameters are set as the above values, respectively, the LDA-RLSURW computes the best AUC on the lncRNADisease dataset. Therefore, we choose the parameters as the corresponding values. For other parameters, we set them as defaults provided by corresponding methods. The proposed LDA-RLSURW method and other comparative methods are evaluated using area under the receiver operating characteristic curve (AUC). Larger AUC values denote better performance.

### 4.2 Performance Comparison With Other Methods

To assess the performance of our proposed LDA-RLSURW method, we compare it with other 10 classical LDA prediction methods, that is, LNCSIM1, LNCSIM2, ILNCSIM, and IDSSIM (Fan W. et al., 2020). LNCSIM1 and LNCSIM2 measured the disease similarity separately using DAGs and the information content and computed association score for each lncRNA-disease pair by Laplacian regularized least squares. IDSSIM designed novel lncRNA functional similarity and disease semantic similarity computation approaches and computed the lncRNA-disease association scores using the computed similarity matrices and weighed K nearest known neighbor method. Table 1 shows the AUC values of LDA prediction methods on the lncRNADisease dataset. From Table 1, we can see that LDA-RLSURW computes the best AUC, which demonstrates the powerful LDA prediction performance of LDA-RLSURW.

**TABLE 1:** AUC values of LDA prediction methods on the lncRNADisease dataset.

	LNCSIM1/LNCSIM2	ILNCSIM	IDSSIM	RWRlncD	IIRWR
5-fold CV	0.8892/0.8881	0.8866	0.8966	0.6976	0.7781
	SIMCLDA	LRLSLDA	LLCPLDA	LDA-LNSUBRW	LDA-RLSURW
	0.7986	0.8174	0.8678	0.8874	0.9027

The LNCSIM1, LNCSIM2, LRLSLDA, and LDA-RLSURW are Laplacian regularized least square-based LDA methods, and the LDA-RLSURW can compute a better AUC. The results demonstrate that integrating unbalanced bi-random random walk can improve the performance. In addition, the IDSSIM and LDA-RLSURW computed the lncRNA similarity and disease similarity using the same method. The IDSSIM used the weighed K nearest known neighbor method to compute the lncRNA-disease association scores. The LDA-RLSURW outperforms IDSSIM, which show that the combination of Laplacian regularized least square and unbalanced bi-random walk can improve the LDA prediction performance compared to weighted K nearest known neighbor method. Both RWRlncD and IIRWR are random walk with restart-based LDA prediction methods. The SIMCLDA is an inductive matrix completion-based method. The LLCPLDA is a locality-constraint linear coding-based method. The LDA-RLSURW computes a better AUC than RWRlncD, IIRWR, SIMCLDA, and LLCPLDA, which further validates the powerful performance of LDA-RLSURW.

### 4.3 Case Study

In this section, we conduct case studies to find potential lncRNA biomarkers for lung neoplasms, NSCLC, and adenocarcinoma of lung after confirming the performance of the proposed LDA-RLSURW method.

#### 4.3.1 Finding Potential lncRNA Biomarkers for Lung Neoplasms

**FIGURE 2:**
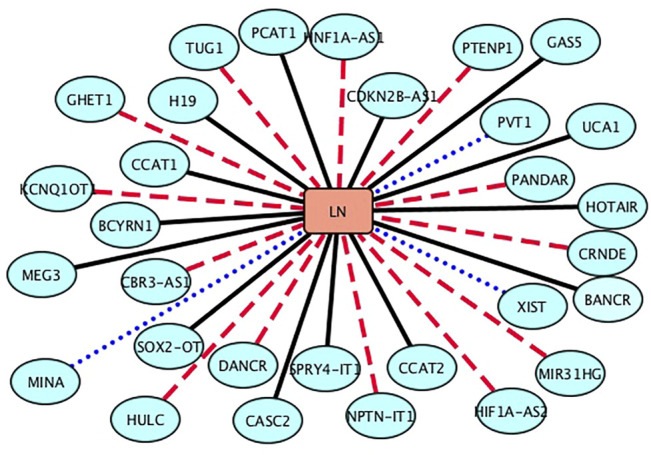
Associations between the inferred top 30 lncRNAs and lung neoplasms (LN). Black solid lines represent known LDAs in the lncRNADisease database. Blue-dot lines represent LDAs that can be observed in the MNDR database. Red-dash lines represent LDAs predicted to be potential lncRNA biomarkers of LN.

Lung neoplasms are one of the leading causes of death associated with malignant tumors in China (Khanmohammadi et al., 2020). Thus, Wang et al. (2020) investigated 14,528 lung cancer patients suffering from multiple primary malignant neoplasms (MPMN) and found 364 MPMN cases. In this section, we inferred the top 30 lncRNA biomarkers associated with lung neoplasms. The results are shown in Table 2 and Figure 2. From Table 2 and Figure 2, we can find that 15 lncRNAs are known to be associated with lung neoplasms in the lncRNADisease database, 3 lncRNAs (MINA, PVT1, and XIST) are unknown to be associated with lung neoplasms in the lncRNADisease database, which can be validated by the MNDR database (Cui et al., 2018). In addition, 12 lncRNAs are predicted to link to lung neoplasms and may be possible biomarkers of lung neoplasms.

**TABLE 2:** Inferred top 30 lncRNAs associated with LN.

Rank	lncRNAs	Evidence	Rank	lncRNAs	Evidence
1	MALAT1	Known	16	MINA	the MNDR database
2	HOTAIR	Known	17	PVT1	the MNDR database
3	MEG3	Known	18	**TUG1**	**Unconfirmed**
4	H19	Known	19	**PANDAR**	**Unconfirmed**
5	GAS5	Known	20	XIST	the MNDR database
6	UCA1	Known	21	**HULC**	**Unconfirmed**
7	CCAT2	Known	22	**HNF1A-AS1**	**Unconfirmed**
8	SPRY4-IT1	Known	23	**PTENP1**	**Unconfirmed**
9	CCAT1	Known	24	**KCNQ1OT1**	**Unconfirmed**
10	CDKN2B-AS1	Known	25	**HIF1A-AS2**	**Unconfirmed**
11	BANCR	Known	26	**DANCR**	**Unconfirmed**
12	BCYRN1	Known	27	**NPTN-IT1**	**Unconfirmed**
13	PCAT1	Known	28	**CRNDE**	**Unconfirmed**
14	SOX2-OT	Known	29	**CBR3-AS1**	**Unconfirmed**
15	CASC2	Known	30	**MIR31HG**	**Unconfirmed**

The bold values denotes lncRNAs that were predicted to associate with LN and need to further validate in Table 2.

More importantly, we predict that lncRNA taurine-upregulated gene 1 (TUG1) may be associated with lung neoplasms. TUG1 is one of lncRNAs that were first identified to associate with human disease. It is linked to diverse physiological processes, for example, gene regulation involved in translation, post-translation, transcription, and post-transcription. In this section, we infer that TUG1 may be the biomarker of lung neoplasms (Guo et al., 2020).

#### 4.3.2 Finding Potential lncRNA Biomarkers for NSCLC

**FIGURE 3:**
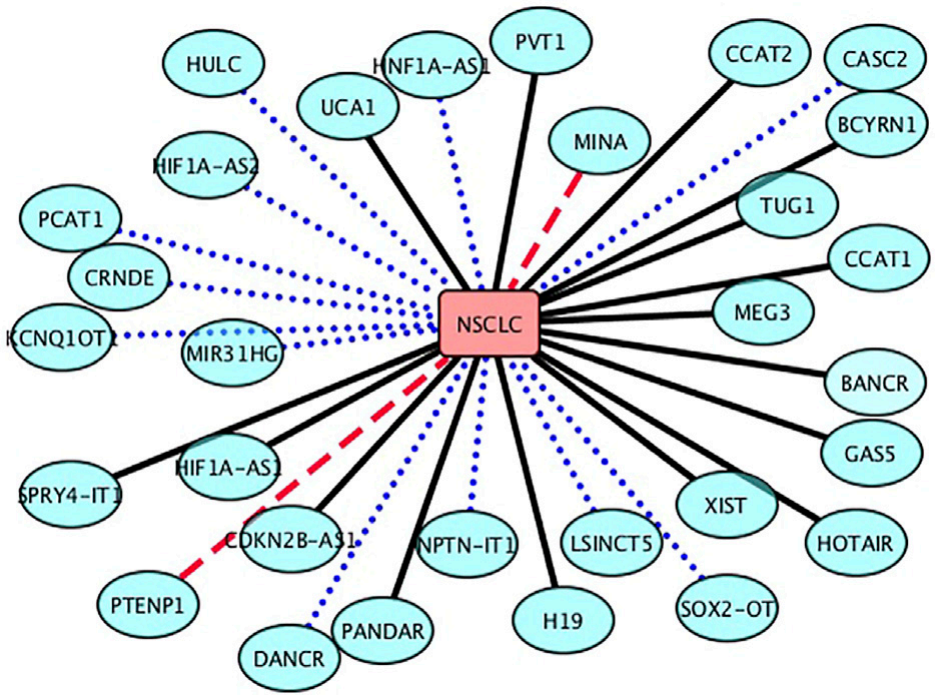
Associations between the inferred top 30 lncRNAs and NSCLC. Black solid lines represent known LDAs in the lncRNADisease database. Blue-dot lines represent LDAs that can be observed in the MNDR database. Red-dash lines represent LDAs predicted to be potential lncRNA biomarkers of LN.

The NSCLC is a subtype of lung cancer. It is one of the leading causes of cancer death in the United States and accounts for 85% of lung cancers among all its subtypes. Although we have achieved important advancements in the NSCLC treatment, our understanding about the biology and mechanisms of NSCLC progression and early detection is still superficial. In this section, we aim to infer new lncRNA biomarkers for NSCLC after confirming the performance of LDA-RLSURW. The predicted top 30 lncRNAs associated with NSCLC are presented in Table 3 and Figure 3. From Table 3 and Figure 3, we can find that 18 lncRNAs associated with NSCLC are known in the lncRNADisease database, 10 lncRNAs associated with NSCLC have been validated in the MNDR database, and 2 lncRNAs (MINA and PTENP1) associated with NSCLC are unknown and require validation. The lncRNA PTENP1 has exerted the tumor-suppressive function through modulating PTEN expression in multiple malignancies. We predict that the PTENP1 may be a potential biomarker of NSCLC (Herbst et al., 2018; Arbour and Riely, 2019; Fan et al., 2020; Leighl et al., 2019).

**TABLE 3:** Inferred top 30 lncRNAs associated with NSCLC.

Rank	lncRNAs	Evidence	Rank	lncRNAs	Evidence
1	MALAT1	Known	16	PANDAR	Known
2	HOTAIR	Known	17	HIF1A-AS1	Known
3	MEG3	Known	18	PCAT1	the MNDR database
4	GAS5	Known	19	CASC2	the MNDR database
5	H19	Known	20	SOX2-OT	the MNDR database
6	UCA1	Known	21	HULC	the MNDR database
7	CCAT2	Known	22	**MINA**	Unconfirmed
8	SPRY4-IT1	Known	23	**PTENP1**	Unconfirmed
9	CDKN2B-AS1	Known	24	HIF1A-AS2	the MNDR database
10	PVT1	Known	25	HNF1A-AS1	Known
11	CCAT1	Known	26	KCNQ1OT1	the MNDR database
12	TUG1	Known	27	CRNDE	the MNDR database
13	BANCR	Known	28	DANCR	the MNDR database
14	BCYRN1	Known	29	MIR31HG	the MNDR database
15	XIST	Known	30	NPTN-IT1	the MNDR database

The bold values denotes lncRNAs that were predicted to associate with NSCLC and need to further validate in Table 3.

#### 4.3.3 Finding Potential lncRNA Biomarkers for Lung Adenocarcinoma

**FIGURE 4:**
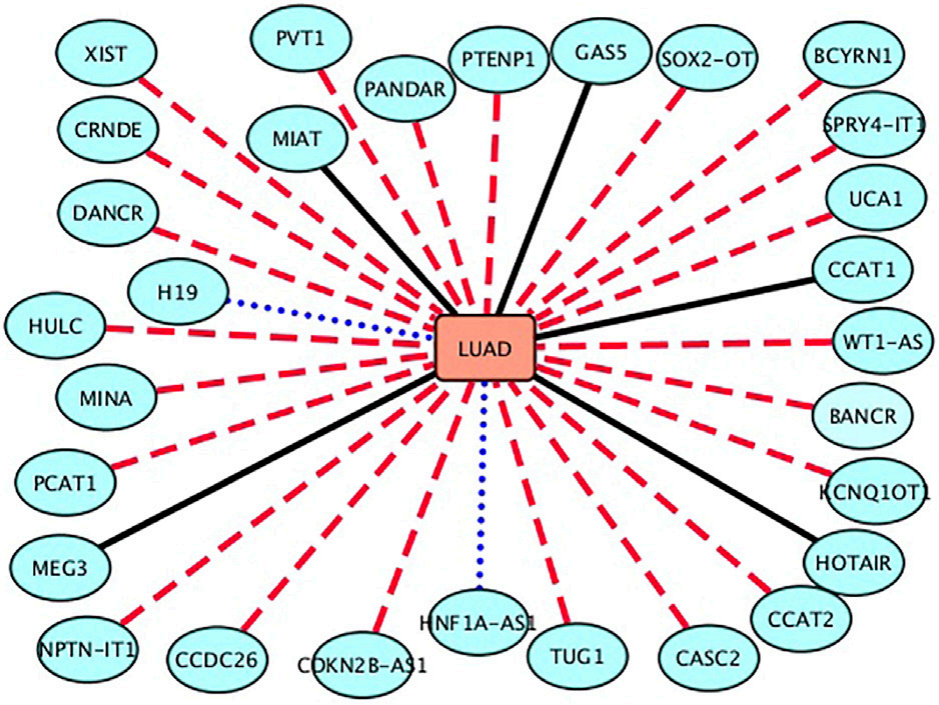
Associations between the inferred top 30 lncRNAs and LUAD. Black solid lines represent known LDAs in the lncRNADisease database. Blue-dot lines represent LDAs that can be observed in the MNDR database. Red-dash lines represent LDAs predicted to be potential lncRNA biomarkers of adenocarcinoma of lung.

The NSCLC is divided into three main subtypes: lung squamous cell carcinoma, large-cell lung cancer, and lung adenocarcinoma (LUAD), among which lung squamous cell carcinoma and LUAD are the most prevalent. In this section, we predict possible lncRNAs associated with LUAD. The results are shown in Table 4 and Figure 4. From Table 4 and Figure 4, we can find that 6 lncRNAs are known to associate with LUAD, 2 lncRNAs are not known to associate with LUAD in the lncRNADisease database, although they are known in the MNDR database, and 22 lncRNAs have not been confirmed to associate with LUAD.

**TABLE 4:** Inferred top 30 lncRNAs associated with LUAD.

Rank	lncRNAs	Evidence	Rank	lncRNAs	Evidence
1	MALAT1	Known	16	**XIST**	Unconfirmed
2	HOTAIR	Known	17	**PANDAR**	Unconfirmed
3	MEG3	Known	18	**BCYRN1**	Unconfirmed
4	GAS5	Known	19	**PCAT1**	Unconfirmed
5	CCAT1	Known	20	**HULC**	Unconfirmed
6	HNF1A-AS1	the MNDR database	21	**CASC2**	Unconfirmed
7	MIAT	Known	22	**SOX2-OT**	Unconfirmed
8	H19	the MNDR database	23	**PTENP1**	Unconfirmed
9	**UCA1**	Unconfirmed	24	**MINA**	Unconfirmed
10	**CDKN2B-AS1**	Unconfirmed	25	**CRNDE**	Unconfirmed
11	**PVT1**	Unconfirmed	26	**DANCR**	Unconfirmed
12	**TUG1**	Unconfirmed	27	**WT1-AS**	Unconfirmed
13	**CCAT2**	Unconfirmed	28	**KCNQ1OT1**	Unconfirmed
14	**SPRY4-IT1**	Unconfirmed	29	**NPTN-IT1**	Unconfirmed
15	**BANCR**	Unconfirmed	30	**CCDC26**	Unconfirmed

The bold values denotes lncRNAs that were predicted to associate with LUAD and need to further validate in Table 4.

Urothelial carcinoma associated 1 (UCA1) is an oncogenic lncRNA. It is highly expressed in many cancers. UCA1 can bind to tumor-suppressive microRNAs, activate a few pivotal signaling pathways, and alter epigenetic and transcriptional regulation. More importantly, its high expression is linked to poor clinicopathological characteristics. In this section, we predict that UCA1 may associate with LUAD and require validation (Yao et al., 2019).

## 5 Discussion

LNCSIM1 and LNCSIM2 obtained better performance improvements based on cross-validation and case analyses. However, LNCSIM1 cannot effectively distinguish the semantic contributions of various disease terms from the identical layer. LNCSIM2 computed the IC values only through integrating DAG information. ILNCSIM is an edge-based prediction model. It combined the concept of information content and the hierarchical structure of DAGs to compute disease semantic similarity.

The RWRlncD conducted random walk with restart on the lncRNA similarity network. However, the RWRlncD cannot be used to predict associated information for diseases without any associated lncRNAs. The IRWRLDA improved random walk-based method through setting an initial probability vector to reduce the disadvantages of random walk with restart. The SIMCLDA used an inductive matrix completion model to complement missing LDA information. The LRLSLDA utilized Laplacian regularized least square model to predict LDAs. The LLCLPLDA first applied a locality-constraint linear coding model to project the local-constraint characteristics of lncRNAs and diseases, and then propagated LDAs by the initial LDA. The LDA-LNSUBRW used linear neighborhood similarity measurement and unbalanced bi-random walk algorithm to find possible LDAs.

The LDA-RLSURW obtains better performance for lncRNA-disease association prediction. It has three advantages: First, it utilizes the biological features to compute the lncRNA and disease similarity. Second, it uses unbalanced bi-random walk to compute the lncRNA-disease association probability. In conclusion, it further computes the lncRNA-disease association probability combining Laplacian regularized least squares.

## 6 Conclusion

Lung cancer is one of the most threatening cancer forms worldwide. In this study, we designed a computational method, LDA-RLSURW, to find possible lncRNA biomarkers for lung cancer. LDA-RLSURW effectively combines unbalanced bi-random walk and Laplacian regularized least square. We predict that TUG1, PTENP1, and UCA1 may be the biomarkers of lung neoplasms, NSCLC and LUAD, respectively.

## Data Availability

The original contributions presented in the study are included in the article/supplementary material. Further inquiries can be directed to the corresponding author.
